# CryEx.v2, an enhanced protocol for the quantitative detection and visualization of cryptic splicing using human transcriptome data

**DOI:** 10.1016/j.xpro.2026.104931

**Published:** 2026-11-06

**Authors:** Rong Zheng, Giovanni Quinones-Valdez, Xinshu Xiao, Chonghui Cheng

**Affiliations:** 1 Lester & Sue Smith Breast Center, Department of Molecular and Human Genetics, Baylor College of Medicine, Houston, TX 77030, USA; 2 Department of Integrative Biology and Physiology and the Molecular Biology Institute, University of California, Los Angeles, Los Angeles, CA 90095, USA

**Keywords:** Bioinformatics, RNA-seq, Gene Expression

## Abstract

Unraveling the mechanisms that suppress cryptic splicing is essential for maintaining transcriptome stability and developing therapeutic strategies in genetic disorders such as cancer. Here, we present a protocol to identify cryptic exons in human epithelial breast cells and quantify their inclusion utilizing RNA sequencing (RNA-seq) data. We describe steps for determining precise chromosome coordinates, calculating percent-spliced-in (PSI) values, classifying exons based on isoform presence, and conducting differential splicing analysis across conditions. We also detail procedures for visualizing a representative splicing event.

For complete details on the use and execution of the original and this updated protocol, please refer to Zheng et al.^1^

## Highlights

• A user-friendly package for CryEx to construct a comprehensive splicing landscape
• Identification and quantification of cryptic and annotated exons using RNA-seq data
• Customizable parameters to enable differential splicing analysis at different resolutions
• A framework for data preprocessing, analysis, customization, and visualization

## Abstract (editor highlights)

Publisher’s note: Undertaking any experimental protocol requires adherence to local institutional guidelines for laboratory safety and ethics.

## Graphical abstract

**Figure:**
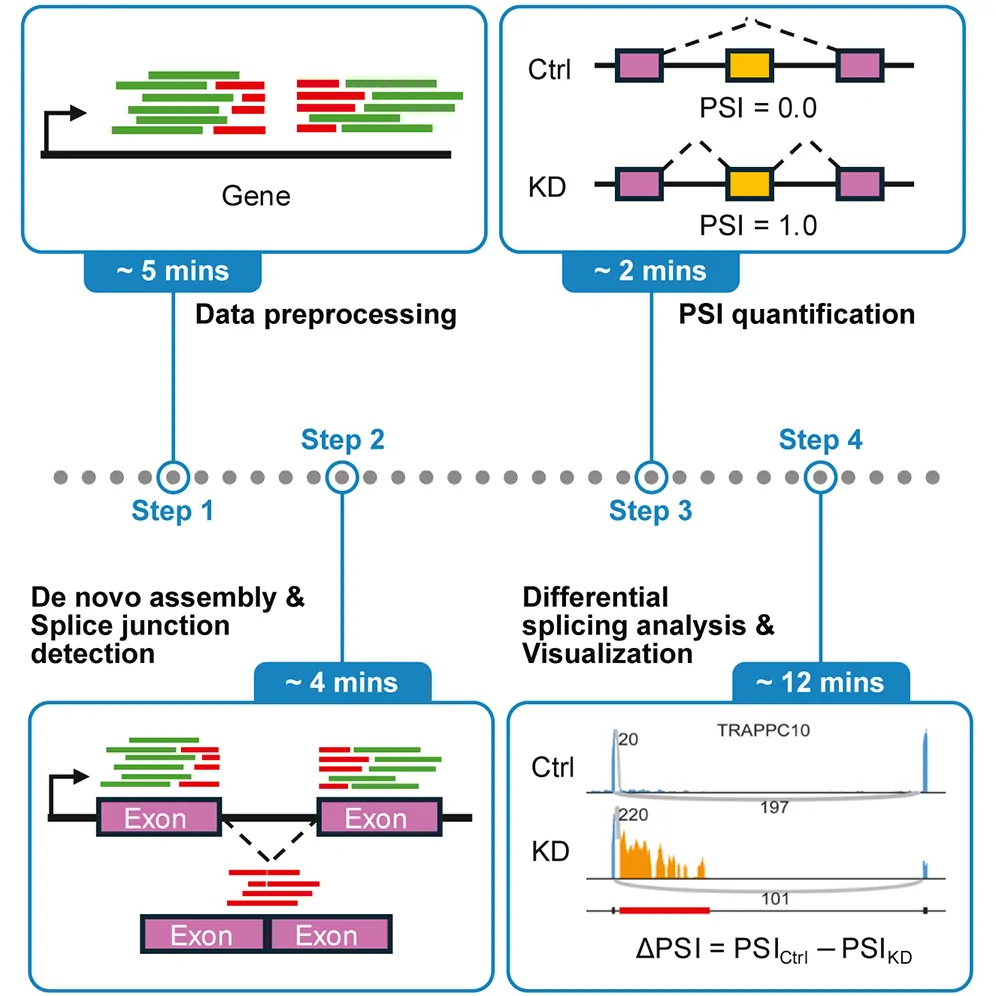

## Before you begin

The human genome contains a high prevalence of pseudo-splice sites, which are typically suppressed under normal conditions. However, abnormal cellular disturbances, such as loss of nuclear protein function,^2^ spliceosome disruption,^3^^,^^4^^,^^5^ or depletion of repressive RNA-binding proteins (RBPs),^6^^,^^7^^,^^8^^,^^9^ can trigger cryptic splicing, potentially contributing to or exacerbating disease pathogenesis. Despite growing interest in cryptic splicing, effective tools for its comprehensive characterization are limited. Classical alternative splicing quantification tools such as rMATS,^10^ SUPPA2,^11^ and MISO^12^ quantify splicing from predefined, annotation-derived transcript models, which restricts detection to canonical, previously annotated events and leaves cryptic, unannotated splicing largely inaccessible to these approaches by design. This gap motivated the development of CryEx^1^ (which refers to cryptic exon) and its updated version, CryEx.v2.

CryEx is a computational pipeline designed to identify and quantify expressed exons, including cryptic and GENCODE-annotated exons, in RNA-seq data. Rather than relying on a fixed reference annotation, CryEx builds an RNA-seq-derived splice junction reference by integrating high-confidence splice junctions from STAR alignments with low-abundance junctions assembled de novo by StringTie under permissive parameters. This strategy enables the detection of rare, unannotated cryptic junctions that annotation-based methods cannot identify. Downstream analysis is then restricted to uniquely mapped reads to ensure specificity. This pipeline constructs a highly customizable table of splicing landscape for each RNA-seq sample, incorporating various filtering parameters, such as precise chromosome coordinates and exon inclusion rates. The table is generated through de novo assembly using RNA-seq reads, detection of splice junctions, and differential PSI quantification for each splicing event per sample, reported in an isoform-specific manner as local three-exon units (upstream, cassette, and downstream exon) rather than as gene-level ratios aggregated across all isoforms—a resolution that distinguishes transcript-specific splicing changes that classical, event-aggregated tools can obscure. In this updated version of the CryEx pipeline, we have developed a Python version that demonstrates augmented and robust performance in identifying and quantifying cryptic exons with significantly reduced runtime.

In this protocol, we provide step-by-step instructions for utilizing the CryEx.v2 Python pipeline to preprocess, identify, quantify, and visualize cryptic exons using RNA-seq data.

### Innovation

Existing splicing calculation tools rely on pre-defined transcript annotations, which restricts exon-inclusion quantification to previously annotated exons and splicing events. In contrast, our pipeline first reconstructs the full set of expressed exons directly from the RNA-seq reads using StringTie, a transcriptome assembly tool, rather than relying solely on a fixed reference annotation. This allows CryEx to detect and quantify splicing of novel exons including cryptic exons.

The application of CryEx.v2 enhances the acquisition of isoform-level localization and precise chromosome coordinates information of cryptic splicing events. It further categorizes cryptic exons by labeling their positions on novel isoforms, offering new insights into their downstream effects on novel peptide fate in cells. This pipeline generates a splicing landscape with intuitive output and customizable parameters, advancing future studies on cryptic splicing.

### Computational requirements for CryEx

1. Computational Requirements:
  a. RAM: Minimum recommended RAM ∼30GB.
  b. Python Version: Minimum required Python version 3.10.0.
  c. STAR: Spliced transcripts alignment to a reference (optional if input files are in BAM format).
  d. StringTie: De novo assembly of RNA-seq alignments into potential transcripts.
  e. Samtools: Alignment manipulation.
  f. Tabix: Index position sorted files in TAB-delimited formats.
  g. Python Modules: In addition to the base Python installation, CryEx.v2 relies on several external Python packages to ensure its functionality and enhance its capabilities. The required external Python packages include:
    i. Multiprocess: allow multiple processors.
    ii. NumPy: mathematical operations on arrays.
    iii. Pandas: data manipulation and analysis.
    iv. Pysam: reading, manipulating, and writing genomic datasets.
    v. Subprocess: creates new child processes.

### Installation of CryEx Python package

**Timing: 5 min**

2. Install the CryEx Python package. Troubleshooting 1.

# PyPI installation:

> pip install CryEx.v2

# GitHub installation:

> git clone https://github.com/giovanniquinones/CryEx

> cd CryEx

> pip install .

# Add to all path:

> export PATH=/installation/path:$PATH

***Note:*** In the above code block, the “#” symbol denotes comments, which are non-executable and serve as annotations within the code.

***Note:*** All code shown is to be ran in bash.

***Note:*** The installation of the CryEx.v2 Python package requires prerequisite dependencies. When installing the package along with its dependencies, the process takes approximately 5 min. However, if the dependencies are already installed, the installation of CryEx.v2 package alone takes less than 1 min.

### Overview of internal data

3. The CryEx.v2 Python package has several annotations and examples of input data within the package. These data objects include:
  a. Transcriptome profile: This dataset contains the chromosome 21 portion of RNA-seq data from mammary epithelial HMLE cells, both with and without *hnRNPM* knockdown, deposited in GEO: GSE227047.
  b. Annotations: GENCODE-backmap GTF annotation and FASTA file from GENCODE Human Release 46 GRCh37

## Key resources table

**Table:** 

REAGENT or RESOURCE	SOURCE	IDENTIFIER
**Software and algorithms**		

STAR (version 2.5.3a)	Dobin et al.^13^	https://github.com/alexdobin/STAR
Python (version 3.10.13)	Python Software Foundation	https://www.python.org
StringTie (version 2.2.1)	Pertea et al.^14^	https://ccb.jhu.edu/software/stringtie
SAMtools (version 1.6)	Danecek et al.^15^	https://www.htslib.org/download
Tabix (version 1.12)	Li et al.^16^	https://github.com/samtools/tabix
multiprocess (version 0.70.16)	Python Standard Library	https://www.python.org
NumPy (version 1.26.4)	Harris et al.^17^	https://pypi.org/project/numpy
pandas (version 2.2.2)	Mckinney et al.^18^	https://pypi.org/project/pandas
pysam (version 0.22.1)	Danecek et al.^15^	https://pypi.org/project/pysam

**Deposited data**		

RNA-seq of HMLE cells expressing control shRNA and *hnRNPM* shRNA	Zheng et al.^1^	Accessible via GEO: GSE227047

**Other**		

Human reference genome primary assembly (GRCh37)	GENCODE Consortium	https://www.gencodegenes.org/human/release_46lift37.html
Human transcriptome GENCODE version 46 backmap to GRCh37 comprehensive assembly	GENCODE Consortium	https://www.gencodegenes.org/human/release_46lift37.html

***Note:*** The CryEx pipeline is agnostic to the genome version and works equally well with GENCODE latest release (GRCh38.p14) https://www.gencodegenes.org/human/

## Step-by-step method details

Here we describe step-by-step details to apply the CryEx.v2 Python package to chromosome 21 bulk RNA-seq data downloaded from GEO: GSE227047 (For demonstration purposes and faster runtime, we restricted the analysis to chromosome 21; however, the CryEx.v2 pipeline is designed to run genome-wide and does not require subsetting). The code is available in the build/lib/CryEx/folder.

### Data preprocessing

**Timing: ∼5 min**

Here, we describe the steps to download and pre-process the data. The steps involve preparing the RNA-seq data and GENCODE annotations using the STAR aligner. These steps ensure that the data is formatted and of sufficient quality for subsequent analysis.

1. Download the RNA-seq FASTQ test data. The test data from our package is deposited in the following repository: https://github.com/giovanniquinones/CryEx/tree/main/test_data. Use the ‘git clone’ command to download.

**Table:** 

SAMPLE	FASTQ	Group
HTE1	fastq/HTE1_chr21_R1.fastq.gz fastq/HTE1_chr21_R2.fastq.gz	ctrl
HTE2	fastq/HTE2_chr21_R1.fastq.gz fastq/HTE2_chr21_R2.fastq.gz	kd
HTE3	fastq/HTE3_chr21_R1.fastq.gz fastq/HTE3_chr21_R2.fastq.gz	ctrl
HTE4	fastq/HTE4_chr21_R1.fastq.gz fastq/HTE4_chr21_R2.fastq.gz	kd

***Note:*** The FASTQ files, containing reads mapped to chromosome 21. Samples HTE1 and HTE3 are derived from control cells, while HTE2 and HTE4 are derived from *hnRNPM* knockdown cells.

***Note:*** The FASTQ test data is paired-end RNA-seq. R1 indicates read1 and R2 indicates read2.

2. Download the gene annotations from GENCODE human database.

***Note:*** The GENCODE-backmap GTF annotation “gencode.v46lift37.annotation.gtf” and its matching genome FASTA file “GRCh37.primary_assembly.genome.fa” from GENCODE consortium are good choices.

3. Perform STAR alignment and generate BAM files. (optional when input files are in BAM format).
  a. Generate a STAR index to use in subsequent alignments.
    > STAR ∖
      --runThreadN 8 ∖
      --runMode genomeGenerate ∖
      --genomeDir STAR_Index ∖
      --genomeFastaFiles GRCh37.primary_assembly.genome.fa ∖
      --sjdbGTFfile gencode.v46lift37.annotation.gtf ∖
      --sjdbOverhang 99
  b. Run STAR alignment on all your sample. Shown is the command with HTE1 sample as input.
    > STAR ∖
      --runThreadN 8 ∖
      --outFilterMultimapNmax 1 ∖
      --outSAMstrandField intronMotif ∖
      --outFilterType BySJout ∖
      --alignSJoverhangMin 8 ∖
      --alignSJDBoverhangMin 3 ∖
      --alignEndsType EndToEnd ∖
      --outSAMtype BAM SortedByCoordinate ∖
      --genomeDir STAR_Index∖
      --readFilesIn ∖
       fastq/HTE1_chr21_R1.fastq ∖
       fastq/HTE1_chr21_R2.fastq ∖
      --outFileNamePrefix bam/HTE1
    ***Note:*** Set the number of threads according to your system's available CPU resources. Additionally, the indexing step requires substantial memory; generating a human genome index typically uses ∼32 GB of RAM across 8 threads. The alignment step memory requirements depend on the number of reads in your FASTQ files. For a typical RNA-seq sample, we recommend at least 32 GB and 8 threads as well. Ensure your system has sufficient memory before running this step.
4. Index the resulting BAM files.

> samtools index bam/HTE1_chr21.bam

5. Generate a tab-separated metadata file ‘fofn.txt’ with information of your samples to be analyzed. It includes the sample name, the full path to the BAM file, the full path to the splice junction file generated by the STAR alignment, and the assigned experimental condition (or group). The format is outlined below:

**Table:** 

SAMPLE	BAM	STAR_SJ_OUT	Group
HTE1	bam/HTE1_chr21.bam	star_sj_out/HTE1_SJ.out.tab	ctrl
HTE2	bam/HTE2_chr21.bam	star_sj_out/HTE2_SJ.out.tab	kd
HTE3	bam/HTE3_chr21.bam	star_sj_out/HTE3_SJ.out.tab	ctrl
HTE4	bam/HTE4_chr21.bam	star_sj_out/HTE4_SJ.out.tab	kd

### *De novo* assembly using StringTie

**Timing: ∼2 min**

To identify any expressed exon in each RNA-seq dataset, we first assemble a highly customized exon annotation based on the RNA-seq reads using StringTie. StringTie is an efficient software that assembles RNA-seq reads into exons and grouping these exons into potential transcripts in an unbiased manner. Next, we detect splice junctions, including their chromosome coordinates and associated junction reads. The StringTie outputs and splice junction files are available in the “out” subfolder. Troubleshooting 2.

6. Run the CryEx_command as indicated below with the metadata file as input and the exons.gtf file as output.

> CryEx_stringtie -f fofn.txt -o out/exons.gtf

***Note:*** The command executed internally is:

> stringtie file.bam -j 2 -f 0.05 -g 10

The output file, ‘exons.gtf’, is a GTF file containing the novel transcripts identified from the input BAM files.

***Note:*** For all CryEx package commands, the ‘--help’ option displays the parameters for each function. For example, you can use ‘CryEx_stringtie --help’ to view the available options and descriptions.

***Note:*** This script assumes the BAM file is paired-end and ‘fr-firststrand’, i.e. the second read in each pair is aligned in the sense direction relative to the RNA. If the data is paired-end and ‘fr-secondstrand’, please use the ‘-s’ option to specify this. For unstranded data, set ‘-s = unstranded’.

### Expressed splice junction detection

**Timing: ∼2 min**

In this step, splice junctions are identified and quantified from the BAM files (denoted by the ‘N’ character in the CIGAR string) for all samples listed in the metadata file ‘fofn.txt’.

7. Run the CryEx_junctions command to identify all expressed splice junctions in the BAM files.

> CryEx_junctions -f fofn.txt -o out/jxn.bed

8. Sort, compress and index the junction bed file for quick lookup.

# Sort, compress, and index junctions BED file

> sort -k1,1V -k2,2n -k3,3n out/jxn.bed | bgzip -c > out/jxn.bed.gz

> tabix -p bed -f out/jxn.bed.gz

***Note:*** The script returns both a regular and a compressed BED file, containing the splice junction coordinates and the number of reads covering each junction for each sample:

**Table:** 

chr21	9907492	9908277	HTE1,HTE2,HTE3,HTE4	63,47,35,49	-
chr21	9907492	9909046	HTE1,HTE2,HTE3,HTE4	0,1,0,3	-
chr21	9908432	9909046	HTE1,HTE2,HTE3,HTE4	12,19,11,8	-

***Note:*** As in Step 2, the default setting for the BAM file is ‘fr-firststrand’. Use ‘-s’ option when the BAM file is not ‘fr-firststrand’.

***Optional:*** If the STAR junction files (“∗SJ.out.tab”) are not provided, the ‘STAR_SJ_OUT’ column in the ‘fofn.txt’ file can be left blank. The junctions will then be identified *de novo* in their entirety.

### Quantify percent-spliced-in for each exon

**Timing: ∼2 min**

To calculate the Percent Spliced In (PSI) for all exons, CryEx.v2 uses the ‘exons.gtf’ files from Step 2 and the splice junctions identified in Step 3. The ‘CryEx_psi_calculator’ tool computes inclusion read counts (inclusion_n), which correspond to RNA-seq reads partially or exclusively aligning to each exon based on the chromosome coordinates in the ‘exon_3ss’ and ‘exon_5ss’ columns. Additionally, it calculates exclusion read counts (exclusion_n) for reads spanning splice junctions containing skipped exons, using the coordinates in the ‘exc_5ss’ and ‘exc_3ss’ columns. PSI is then determined using the inclusion and exclusion values, as follows:

$$PSI=\frac{\frac{inc}{RL+XL}}{\frac{inc}{RL+XL}+\frac{exc}{RL}}$$

***Note:*** Here, ‘inc’ and ‘exc’ represent the ‘inclusion_n’ and ‘exclusion_n’ read counts, respectively. RL refers to the length of the individual RNA-seq read, while XL denotes the length of the variable exon or retained intron.

9. Run the CryEx_psi_calculator command:

> CryEx_psi_calculator ∖

 -f fofn.txt ∖

 -e out/exons.gtf ∖

 -j out/jxn.bed.gz ∖

 -o out/psi.txt

***Note:*** The ‘psi.txt’ file output contains the following fields:

**Table:** 

exon_type	chrom	exon_3ss	exon_5ss	strand	inclusion_n
first_exon	chr21	9907191	9907492	-	127
first_exon	chr21	9907191	9907492	-	97
first_exon	chr21	9907191	9907492	-	67

**Table:** 

exc_5ss	exc_3ss	exclusion_n	SAMPLE	PSI
9896772	9966321	0	HTE1	1.00
9896772	9966321	1	HTE2	0.96
9896772	9966321	0	HTE3	1.00

***Note:*** As in Step 2, the default setting for the BAM file is ‘fr-firststrand’. The ‘-s’ option should be used if the BAM file is not ‘fr-firststrand’. Additionally, the required input file for this step is the compressed and indexed BED file generated in Step 3.

### Perform differential splicing analysis

**Timing: ∼2 min**

By utilizing the ‘Group’ information from the metadata file, individual samples can be organized into conditions or groups, allowing for the calculation of differential splicing, represented by ‘DeltaPSI’ for each exon.

10. Run the CryEx_diff command:
  > CryEx_diff -f fofn.txt -p out/psi.txt -o out/diff.txt
  ***Note:*** We employ a beta-binomial approach to test the null hypothesis that the underlying splicing ratio for each exon is the same between the two groups. This approach is adapted from the study by Tran et al.^19^ In brief, for each exon *i*, we calculate the probability of the observed PSI based on the hyperparameters alpha and beta.
  $$P\left(inc_{i},PSI_{i}\left|n_{i},\alpha_{1},\beta_{1})\;∼\;Binomial(k_{i}\right|n_{i},PSI_{i}\right)\times Beta\left(PSI_{i}\;|\alpha_{1},\beta_{1}\right)$$
  We then calculate the Likelihood of the PSI values assuming both groups have the same underlying splicing ratio i.e., the same alpha and beta values.
  $$L_{0}=\prod_{1}^{m}P\left(inc_{i},PSI_{i}|n_{i},\alpha_{0},\beta_{0}\right)$$
  The alternate hypothesis is that samples from group 1 (samples 1 to j) have different underlying hyperparameters from group 2 (j to m).
  $$L_{A}=\prod_{1}^{j}P\left(inc_{i},PSI_{i}|n_{i},\alpha_{1},\beta_{1}\right)\times \prod_{j}^{m}P\left(inc_{i},PSI_{i}|n_{i},\alpha_{2},\beta_{2}\right)$$
  The values of alpha and beta are obtained using the mean (*μ*) and standard deviation (*σ*) of the PSI values for each group, respectively:
  $$\alpha =\frac{\mu }{\sigma };\;\beta =\frac{1-\mu }{\sigma }$$
  The log-likelihood ratio is given as follows:
  $$LLR=\log\frac{L_{A}}{L_{0}}$$
  Based on Wilk’s theorem, the statistical significance of this test is given by the formula:
  $$2\times LLR\;∼\;\chi^{2}$$
  ***Note:*** The output file has the following format (3 of 5 differential events shown for chromosome 21):

**Table:** 

exon_type	exon_coords	flanking_jxns	LLR	Pvalue	Sig
last_exon	chr21:45458057-45462429:+	45457808,45472160	9.6	6.78e-05	True
first_exon	chr21:45514756-45516594:+	45514114,45518237	8.32	2.43e-04	True
middle_exon	chr21:45516486-45516594:+	45514114,45518237	8.32	2.43e-04	True
  **Table:** 

DeltaPSI	DIFF	PSIGroup1	PSIGroup2	CovGroup1	CovGroup2	Pvalue_Adj
-0.54	ctrl-kd	0.01,0.02	0.56,0.54	238,219	285,323	1.16e-02
-0.26	ctrl-kd	0.02,0.02	0.20,0.36	206,210	74,50	1.65e-02
-0.48	ctrl-kd	0.07,0.07	0.48,0.62	206,210	74,50	1.65e-02
  The identified exons are primarily categorized into several types: **1) first_exon:** the cryptic exon is the first exon of the transcript and contains only the 5′ splice site; **2) middle_exon:** the cryptic exon is located in the middle of the transcript and includes both 5′ and 3′ splice sites; **3) last_exon:** the cryptic exon is the last exon of the transcript and contains only the 3′ splice site; **4) retained intron:** the coordinates corresponds to a retained intron; **5) not_spliced**: no splice junction reads are detected.
  ***Note:*** The fields ‘exon_type’, ‘exon_coords’, and ‘flanking_junctions’ are used to identify individual splicing events, retaining the same information from the previous step. The ‘LLR’ and ‘Pvalue’ represent the log-likelihood ratio and p-value, respectively, from the differential splicing test. The label ‘Sig’ indicates whether the event is considered differential based on established cutoffs (See the Optional section). ‘DeltaPSI’ denotes the difference in mean PSI values between the two groups, with ‘DIFF’ indicating the direction of the difference. In this example, we subtract the mean PSI of the ‘kd’ samples from the mean PSI of the ‘ctrl’ samples. The coverage value columns represent the total number of reads at each splice junction, plus reads aligning to the identified exon, for each individual sample. The PSI value columns contain the calculated PSI for each event in the individual samples.
  ***Note:*** For cryptic splicing analyses, annotated splicing events are subsequently removed, and events with PSI ≥ 0.1 in control samples are also excluded to enrich for aberrant cryptic events with low background inclusion in normal cells.
  ***Optional:*** Users can adjust the stringency of the test by specifying the following parameters:
  a. --min_llr: The minimum Log-Likelihood Ratio (LLR) value required to classify an event as significant. The default value is 2.
  b. --min_cov: The minimum coverage required for every sample for the event to be considered testable. The default value is 5 reads.
  c. --min_delta_psi: The minimum absolute difference in mean PSI values between the two groups. The default value is 0.10.

### Visualization of representative splicing event

**Timing: ∼10 min**

To generate a customizable sashimi plot of a splicing event of interest, you can use the package ‘ggsashimi’.

11. Reformat the metadata file fofn.txt to match the format required by ggsashimi (columns: sample ID, path to BAM file, group/category; no column headers).

> cat test_data/fofn.txt | grep -v SAMPLE | cut -f1,2,4 > input_bams.tsv

12. Define the precise chromosomal coordinates of the target exon, along with its adjacent upstream and downstream exons, and providing a GTF file (here, the GTF generated by StringTie) to plot the transcript structures.

> GTF=test_data/out/exons.gtf

> Interval=“chr21:45457673-45472222”

13. Generate the sashimi plot with the ggsashimi.py script, specifying aesthetic parameters as preferred. Installation and usage of ggsashimi^20^ is documented in its GitHub repository: https://github.com/guigolab/ggsashimi. Here, we use the -C 1 and -O 1 options to use the first column of input_bams.tsv for coloring and grouping, respectively. All other parameters are optional. For this demonstration, we focus on a representative cryptic exon located on chromosome 21 (chr21:45457673-45472222), as shown in Figure 1. Adobe Illustrator was used to polish and label the figure below.

**Figure 1:**
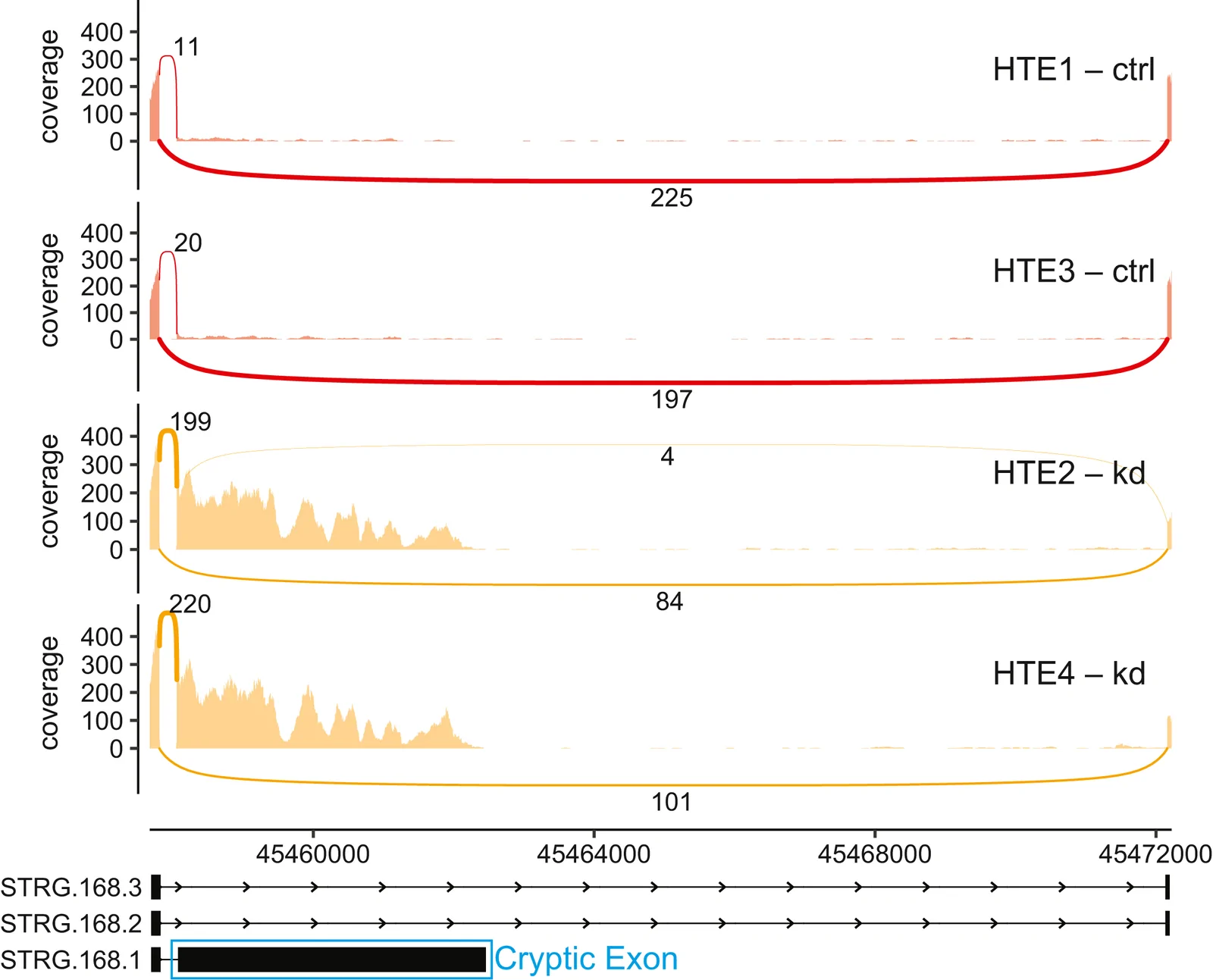
Sashimi plot visualization of the cryptic exon in the *TRAPPC10* gene Numbers of splice junction reads are shown next to arches.

# Custom color palette

> printf “red∖norange∖n” > palette.txt

# Run ggsashimi with desired aesthetics

> ./ggsashimi.py ∖

  -b input_bams.tsv ∖

  -g ${GTF} ∖

  -c ${interval} ∖

  --base-size=14 ∖

  --height=1.5 ∖

  --ann-height=3 ∖

  --width=7 ∖

  --fix-y-scale ∖

  -P palette.txt ∖

  -M 3 ∖

  -C 1 ∖

  -O 1 ∖

  -F pdf

***Note:*** After using graphics edit tools such as Illustrator to polish the figure above, the refined IGV plot can be referenced in Figure 2C of Zheng et al. (2024).^1^

## Expected outcomes

The CryEx.v2 enables the annotation and quantification of cryptic exons from transcriptome profile. The CryEx.v2 output provides accurate and detailed information, including 1) precisely defined 3′ and 5′ splice sites of any expressed exon; 2) upstream and downstream flanking exons; 3) exon type classified based on the exon’s position within the isoform (First, Middle, Last and Retained Intron); 4) inclusion rate measured by PSI; and 5) differential splicing changes (ΔPSI) across conditions, along with statistical significance. The CryEx.v2 output table is highly customizable, allowing for additional parameters such as the number of reads at splice site, number of reads aligned to expressed exons, and the number of reads in junctions, among others.

## Limitations

The current CryEx.v2 pipeline primarily focuses on identifying cryptic splicing events, including cryptic first exons, middle exons, and last exons, and retained introns. Future versions will expand the scope to capture additional types of cryptic events, such as exitrons,^21^ alternative 5′ splice site (5′SS), alternative 3′ splice site (3′SS), mutually exclusive exons, and alternative polyadenylation (APA), providing a more comprehensive understanding of the cryptic splicing landscape.^21^

## Troubleshooting

### Problem 1

It fails to ‘pip install’ and returns this error message “module ‘pip._vendor.platformdirs’ has no attribute ‘user_cache_dir’”.

### Potential solution

• Try ‘pip3 install’ instead of ‘pip install’.

### Problem 2

The script fails when the following command is executed: ‘CryEx_stringtie -f fofn.txt -o exons.gtf’ and returns a “Segmentation fault” error message.

### Potential solution

• Run StringTie on each file one by one instead of multiple files in one run. Then merge the output into one final GTF file.

## Resource availability

### Lead contact

Further information and requests for resources and reagents should be directed to and will be fulfilled by the lead contact, Chonghui Cheng (chonghui.cheng@bcm.edu).

### Technical contact

Technical questions on executing this protocol should be directed to and will be answered by the technical contact, Giovanni Quinones-Valdez (giovas@ucla.edu) and Rong Zheng (rongzheng92@gmail.com).

### Materials availability

This study did not generate any new unique reagents.

### Data and code availability

The datasets generated during this study are available at GEO: GSE227047. The code generated during this study is available at GitHub: https://github.com/giovanniquinones/CryEx. The specific version 2.0.1 in this protocol for the CryEx Python package is also archived in Zenodo: https://zenodo.org/uploads/15500203.

## Acknowledgments

This research was supported in part by grants from R01CA262686 and R01AG078950 (to X.X.) and R35GM131876 (to C.C.). C.C. is a 10.13039/100004917Cancer Prevention Research Institute of Texas (CPRIT) Scholar in Cancer Research (RR160009 to C.C.).

## Author contributions

Conceptualization, X.X., C.C., R.Z., and G.Q.-V.; software, G.Q.-V. and R.Z.; formal analysis, G.Q.-V.; investigation, writing, review, and editing, G.Q.-V. and R.Z.; visualization, G.Q.-V. and R.Z.; supervision, X.X. and C.C.; funding acquisition, X.X. and C.C.; correspondence and code maintenance, G.Q.-V.

## Declaration of interests

The authors declare no competing interests.

## Declaration of generative AI and AI-assisted technologies in the manuscript preparation process

During the preparation of this work, the authors used ChatGPT to improve the quality of the writing by correcting typos and grammar errors and refining sentence structures. After using this tool, the authors reviewed and edited the content carefully and take full responsibility for the final content of the publication.
